# Conserved cholesterol-related activities of Dispatched 1 drive Sonic hedgehog shedding from the cell membrane

**DOI:** 10.1242/jcs.258672

**Published:** 2021-08-19

**Authors:** Kristina Ehring, Dominique Manikowski, Jonas Goretzko, Jurij Froese, Fabian Gude, Petra Jakobs, Ursula Rescher, Uwe Kirchhefer, Kay Grobe

**Affiliations:** 1 Institute of Physiological Chemistry and Pathobiochemistry, University of Münster, Waldeyerstrasse 15, D-48149 Münster, Germany; 2 Center for Molecular Biology of Inflammation, Institute for Medical Biochemistry, University of Münster, Von Esmarch Strasse 56, D-48149 Münster, Germany; 3 Institute of Pharmacology and Toxicology, University of Münster, Domagkstrasse 12, D-48149 Münster, Germany

**Keywords:** Hedgehog, Shedding, Cholesterol, Patched, Dispatched, Resistance–nodulation–division, Sterol-sensing domain

## Abstract

The Sonic hedgehog (Shh) pathway controls embryonic development and tissue homeostasis after birth. Long-standing questions about this pathway include how the dual-lipidated, firmly plasma membrane-associated Shh ligand is released from producing cells to signal to distant target cells and how the resistance–nodulation–division transporter Dispatched 1 (Disp, also known as Disp1) regulates this process. Here, we show that inactivation of Disp in Shh-expressing human cells impairs proteolytic Shh release from its lipidated terminal peptides, a process called ectodomain shedding. We also show that cholesterol export from Disp-deficient cells is reduced, that these cells contain increased cholesterol amounts in the plasma membrane, and that Shh shedding from Disp-deficient cells is restored by pharmacological membrane cholesterol extraction and by overexpression of transgenic Disp or the structurally related protein Patched 1 (Ptc, also known as Ptch1; a putative cholesterol transporter). These data suggest that Disp can regulate Shh function via controlled cell surface shedding and that membrane cholesterol-related molecular mechanisms shared by Disp and Ptc exercise such sheddase control.

## Abstract (precis)

**Summary:** The resistance–nodulation–division protein Dispatched 1 expels plasma membrane cholesterol to indirectly increase Sonic hedgehog morphogen release from the cell surface.

## INTRODUCTION

Hedgehog (Hh) ligands activate an evolutionarily conserved signaling pathway that provides instructional cues during tissue morphogenesis and, if misregulated, can contribute to developmental disorders and cancer. Fully bioactive Hh is posttranslationally modified by a cholesteryl moiety at the C terminus (Porter et al., 1996) and a palmitoyl group at the N terminus (Pepinsky et al., 1998). Both lipids firmly tether Hh to the plasma membrane of the producing cell to effectively prevent unregulated ligand release. Signaling at distant cells therefore requires regulated Hh removal from the membrane, a process that is facilitated by vertebrate and invertebrate Dispatched 1 (Disp, also known as Disp1) orthologs: genetic studies in flies and mice have revealed that Disp is specifically required in Hh ligand-producing cells and that Disp inactivation reduces ligand release and compromises Hh pathway activity *in vivo* (Burke et al., 1999; Kawakami et al., 2002; Ma et al., 2002; Nakano et al., 2004). Yet, the mechanistics of Disp-dependent Hh release remained unclear. Long-lasting questions about the Hh pathway are therefore (1) how Disp drives dual-lipidated Hh release from the plasma membrane, (2) whether Disp acts directly or indirectly in the process, and (3) to what carrier – if any – Hh is transferred.

What makes these questions particularly interesting is that the Hh release protein Disp on Hh-producing cells is structurally related to the Hh receptor Patched 1 (Ptc, also known as Ptch1) on Hh-receiving cells (Hall et al., 2019). Both proteins contain 12 transmembrane helices and two extracellular domains and belong to the resistance–nodulation–division (RND) family of transmembrane efflux pumps. In addition, both proteins contain a conserved domain known as the sterol-sensing domain (SSD) that is involved in different aspects of homeostasis of free or esterified cellular cholesterol in other SSD proteins (Hall et al., 2019). These striking structural resemblances between Ptc and Disp and the conserved SSD constitute further unexplained features of the Hh pathway, because they imply that similar – possibly cholesterol related – mechanisms control the opposite functions of Hh release from producing cells and Hh perception at receiving cells.

In this study, to characterize Disp-dependent release of the vertebrate Hh family member Sonic hedgehog (Shh) from the plasma membrane, we produced murine Shh in Bosc23 cells, a derivative of HEK293 cells that endogenously express Disp (Jakobs et al., 2014). Notably, in our *in vitro* system, we made sure that Shh biosynthesis faithfully undergoes all required posttranslational modifications to generate the dual-lipidated, fully bioactive plasma membrane-associated morphogen. The first posttranslational modification consists of the removal of the Shh signal sequence during translocation into the endoplasmic reticulum. The resulting 45 kDa precursor proteins consist of an N-terminal signaling domain that starts with a cysteine (C25 in mouse Shh) and a C-terminal autoprocessing/cholesterol transferase domain. For the second modification, the autoprocessing/cholesterol transferase domain covalently attaches cholesterol to the C terminus of the N-terminal signaling domain and simultaneously splits the 45 kDa precursor protein at the cholesteroylation site (Bumcrot et al., 1995) to ensure complete C-terminal cholesteroylation of all Shh signaling domains. In contrast, the third essential posttranslational Hh modification – N-lipidation of signaling domains – requires a separate enzymatic activity encoded by the Hh palmitoyltransferase Hhat, the lack or insufficient expression of which results in the secretion of non-palmitoylated inactive Shh (Chamoun et al., 2001). Because HEK293 and Bosc23 cells lack sufficient endogenous Hhat activity (Jakobs et al., 2014), throughout this work, we expressed the 45 kDa Shh precursor together with human Hhat from one bicistronic mRNA (Jakobs et al., 2014). We then compared dual-lipidated Shh release from the plasma membrane of Disp-expressing and Disp-deficient Bosc23 cells using SDS–PAGE and immunoblotting. We found that Disp regulates proteolytic Shh processing from both lipidated membrane anchors (another posttranslational modification called shedding), because Shh shedding from Disp-deficient cells was strongly and specifically reduced when compared to Disp-expressing control cells. [^3^H]-cholesterol efflux assays further revealed that Disp-deficient cells are impaired in their ability to secrete [^3^H]-cholesterol into the culture medium, and cholesterol quantification assays showed that the amounts of free membrane cholesterol in these cells are significantly increased. These findings suggest that the primary function of Disp is to control the amount or spatial distribution of membrane cholesterol at the cell surface, and that cholesterol-dependent physical properties of the plasma membrane may in turn control Shh shedding. We support this possibility by demonstrating restored Shh shedding from Disp-deficient Bosc23 cells upon pharmacological cholesterol depletion or overexpression of the putative cholesterol pump Ptc (Zhang et al., 2018). These data link the known structural conservation between Disp and Ptc with a shared membrane cholesterol-related mechanism that is essential for both Hh perception in target cells and – as shown in this study – Hh relay from producing cells.

## RESULTS

**Fig. 1.**
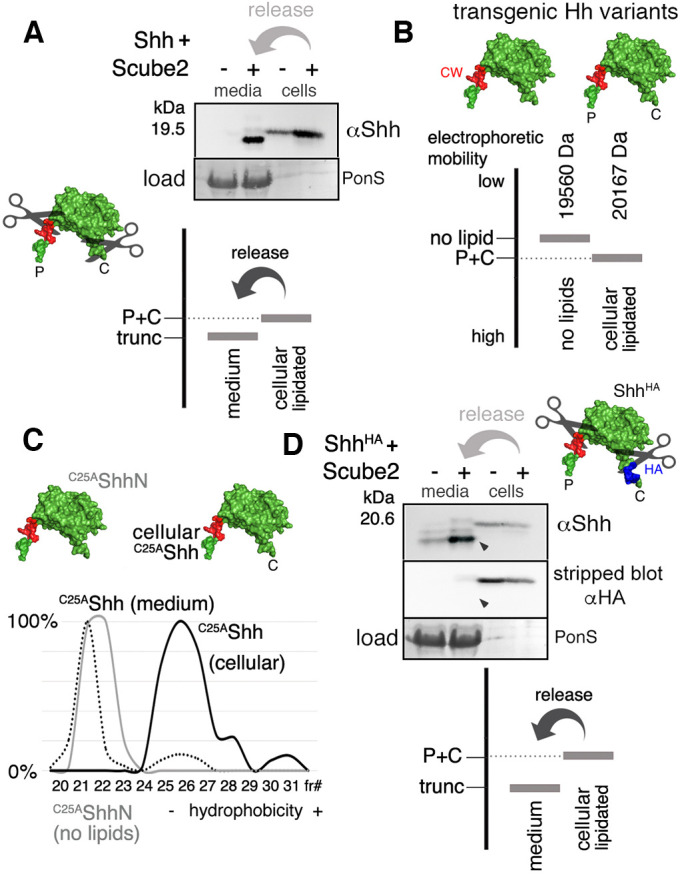
**Scube2 enhances proteolytic Shh processing.** (A–D) Schematics of expressed Shh constructs are shown, using PDB:3M1N as a template. P, palmitate; C, cholesterol; CW, Cardin–Weintraub motif representing the N-terminal cleavage site; scissors indicate loss of lipidated terminal peptides during release from Bosc23 cells. CW is shown in red; an inserted HA tag is shown in blue. (A) Increased electrophoretic mobility of soluble Shh (trunc) over the dual-lipidated cellular protein (P+C) results from the loss of both lipidated terminal peptides during release from Bosc23 cells (indicated by scissors), and this process depends on Scube2. Cellular (cells) and released (media) proteins were detected using Shh-specific antibodies (αShh). Residual serum albumin in TCA-precipitated supernatants served as loading control (PonceauS staining, PonS). Bottom: schematic of Shh release. (B) According to previous publications (Pepinsky et al., 1998; Porter et al., 1996), dual-lipidated Hh (cellular lipidated) migrates faster in SDS–PAGE than *E. coli*-expressed unlipidated Hh (no lipids), although mass spectrometry has determined molecular masses of 20,167 Da for the former form and 19,560 Da for the latter. Increased electrophoretic lipidated Hh mobility, despite higher molecular mass, is caused by SDS association with the large hydrophobic sterol backbone of cholesterol and the C_16_ hydrocarbon tail of the palmitate. Consistent with this, chemical hydrolysis of the ester bond that attaches cholesterol to Shh decreases the electrophoretic mobility of the delipidated product (Zeng et al., 2001). The observed increase in dual-lipidated Shh electrophoretic mobility during release from human cells (as shown in A) can therefore only result from the additional loss of associated terminal peptides, which more than compensates for this decrease. (C) Reverse-phase HPLC. The elution profile of Bosc23-expressed non-lipidated control ^C25A^ShhN (gray line) resembles that of soluble ^C25A^Shh (dotted line), but not that of its lipidated cellular precursor (black solid line). Elution profiles are expressed relative to the highest protein amount in a given fraction (set to 100%). fr#, fraction number. (D) Insertion of a C-terminal HA tag supports shedding. Removal of terminal peptides including the 1 kDa C-terminal tag increases the net electrophoretic mobility gain of solubilized proteins (arrowhead) (Jakobs et al., 2014, 2017), as visualized using antibodies against Shh and the HA tag (αHA). Data in A,C,D are representative of at least three independent experiments.

In the first part of our study, we established essential *in vitro* conditions for the release of physiologically relevant Hh from Disp-expressing cells into serum-depleted medium (Creanga et al., 2012; Jakobs et al., 2014; Tukachinsky et al., 2012). First, we expressed Shh together with Hhat to minimize the production of non-palmitoylated or only partially palmitoylated overexpressed Shh, as described previously (Jakobs et al., 2014). Second, it is known that solubilization of the dual-lipidated vertebrate Hh family member Shh from the plasma membrane requires a synergistic factor called Scube2 [signal peptide, cubulin (CUB) and epidermal growth factor (EGF)-like domain-containing protein 2; Hall et al., 2019]. Scube2 activity in Shh release critically depends on its C-terminal CUB domain (Creanga et al., 2012; Tukachinsky et al., 2012), which derives its name from the complement subcomponents C1r and C1s, sea urchin protein with EGF-like domains (UEGF) and bone morphogenetic protein 1 (BMP1). CUB domains contribute to protease activities in these proteins (Gaboriaud et al., 2011), possibly by binding to and inducing structural changes in the substrate to boost turnover (Bourhis et al., 2013; Jakobs et al., 2017). Alternatively, Scube2 has been implicated in the transfer of dual-lipidated Shh from Shh-expressing cells to distant receiving cells (Tukachinsky et al., 2012; Wierbowski et al., 2020). To distinguish between these possibilities, we produced dual-lipidated Shh in Disp-expressing Bosc23 cells in the presence or absence of Scube2, and analyzed cellular and solubilized proteins using SDS–PAGE and immunoblotting. Our analyses confirmed previously published reports (Jakobs et al., 2014, 2016) that Scube2 strongly enhances Shh solubilization and revealed increased electrophoretic mobility of most released Shh over that of the corresponding dual-lipidated cellular material (Fig. 1A). Such increased electrophoretic Shh mobility can be best explained by the proteolytic removal of both lipids together with the associated terminal peptides during release for three main reasons. First, the removal of Shh lipids alone, for example by chemical saponification, decreases electrophoretic Shh mobility instead of increasing it (Porter et al., 1996) (schematic in Fig. 1B). This rules out Shh release by hypothetical esterase equivalents. Second, reverse-phase high-performance liquid chromatography (HPLC) directly confirmed lipid loss during Shh release, because, in the presence of Scube2, a cholesterol-modified but non-palmitoylated cellular ^C25A^Shh variant (N-terminal cysteine replacement by a serine or an alanine blocks Shh palmitoylation; Hardy and Resh, 2012) converted into less hydrophobic soluble ^C25A^Shh (Fig. 1C). Third, Scube2 enhanced the conversion of hemagglutinin (HA)-tagged dual-lipidated Shh^HA^ (in this construct, the HA tag was inserted adjacent to the cholesteroylated glycine 198) into truncated proteins that lacked the tag (Fig. 1D). These findings strongly suggest near-complete Shh delipidation during sheddase-mediated release, which is in line with previous *in vitro* (Jakobs et al., 2014, 2017) and *in vivo* (Palm et al., 2013; Schürmann et al., 2018) observations.

**Fig. 2.**
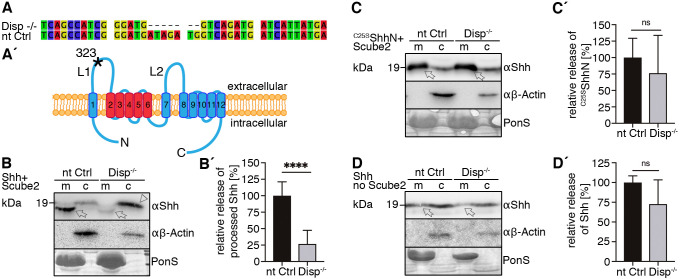
**Impaired Shh release from Disp^−/−^ cells.** (A) Alignment of targeted *disp* gene sequences from Disp^−/−^ cells and from non-targeted (nt Ctrl) cells. (A′) Schematic representation of the Disp protein structure. An asterisk indicates the CRISPR/Cas9-generated stop codon introduced at position 323, deleting 11 of 12 TM domains that together represent ∼80% of the protein sequence. L1 and L2 indicate extracellular loops. TM2–TM6 (colored red) constitute the SSD. (B,C) Immunoblots of cellular (c) and released (into the medium, m) Shh (B) and unlipidated control ^C25S^ShhN (C) in nt Ctrl and Disp^−/−^ cells in the presence of Scube2. Arrows indicate solubilized Shh and the arrowhead indicates accumulated cellular material in Disp^−/−^ cells. (D) In the absence of Scube2, Shh processing into serum-free medium was abolished in nt Ctrl and Disp^−/−^ cells. Instead, both cell types released similar amounts of unprocessed protein. In B,C,D, anti-β-actin blots (αβ-actin) and Ponceau S staining of residual serum albumin (PonS) serve as loading controls. (B′,C′,D′) Quantifications of relative Shh (B′,D′) and ^C25S^ShhN (C′) release from nt Ctrl and Disp^−/−^ cells. Ratios of solubilized versus cellular Shh were determined and expressed relative to Shh release from nt Ctrl cells (black bars). Data are mean±s.d. *n*=21 in B′, *n*=8 in C′ and *n*=5 in D′. ****P<0.0001; ns, not significant (two-tailed unpaired *t*-test).

Based on these findings, and because Scube2-regulated Hh solubilization is known to require Disp (Hall et al., 2019), we expected strongly and specifically impaired Shh shedding from cells made deficient in Disp function. To test this hypothesis, we generated Disp-knockout cells (Disp^−/−^) using CRISPR/Cas9 genome editing in Bosc23 cells. Sequencing of the targeted genomic loci confirmed deletion of 7 base pairs, leading to a frameshift and a stop codon at amino acid 323 located in the first extracellular loop of the 1524-amino-acid protein (Fig. 2A,A′). We verified that predicted off-target sites were unaffected (Table S1) and confirmed complete Disp protein loss in Disp^−/−^ cells using immunoblotting (Fig. S1A). Transgenic overexpression of murine Disp (Disp^tg^) restored the immunoblot signal, demonstrating effectivity and specificity of the targeting approach (Fig. S1A′). We then analyzed Shh release from the plasma membrane of Disp- expressing and Disp-deficient Bosc23 cells into the supernatant using SDS–PAGE and immunoblotting. Consistent with the findings shown in Fig. 1A, Shh was released from Disp-expressing *CRISPR* non-targeting control cells (nt Ctrl) in the presence of Scube2, and the electrophoretic mobility of most released Shh was increased over that of the corresponding dual-lipidated cellular material (Fig. 2B, arrow). Notably, Shh shedding from Disp^−/−^ cells was significantly reduced compared to that from nt Ctrl cells (Fig. 2B, arrows, B′). Instead, Disp^−/−^ cells accumulated cellular Shh (Fig. 2B, arrowhead), as has been shown *in vivo* (Burke et al., 1999). Also consistent with previous observations (Kawakami et al., 2002; Ma et al., 2002), loss of Disp in Bosc23 cells did not affect Shh biosynthesis or autoprocessing of the primary translation product into the 19 kDa cholesteroylated signaling domain (Fig. S1B). Disp loss also did not affect morphogen secretion into serum-depleted medium, because unlipidated control ^C25S^ShhN (having the palmitate-accepting cysteine replaced and also lacking the C-terminal autoprocessing/cholesterol transferase domain) was readily released (Fig. 2C, arrows, C′). We conclude that Disp increases shedding of dual-lipidated Shh into bioactive truncated proteins (Fig. S2A,B), but that Disp is not essential for this process per se, because small Shh amounts can also be released from Disp^−/−^ cells (Fig. 2B, arrow). This supports previous observations of non-essential Disp function for Indian Hh signaling in the skeleton (Tsiairis and McMahon, 2008) and in cells or tissues that generate high levels of Hh (Nakano et al., 2004). Notably, in the absence of Scube2 (Fig. 2D,D′), apparently unprocessed Shh (as indicated by similar electrophoretic mobilities of cellular and soluble proteins; Fig. 2D, arrows) is somehow released in a Disp-independent manner, suggesting that the underlying mechanism is physiologically irrelevant.

**Fig. 3.**
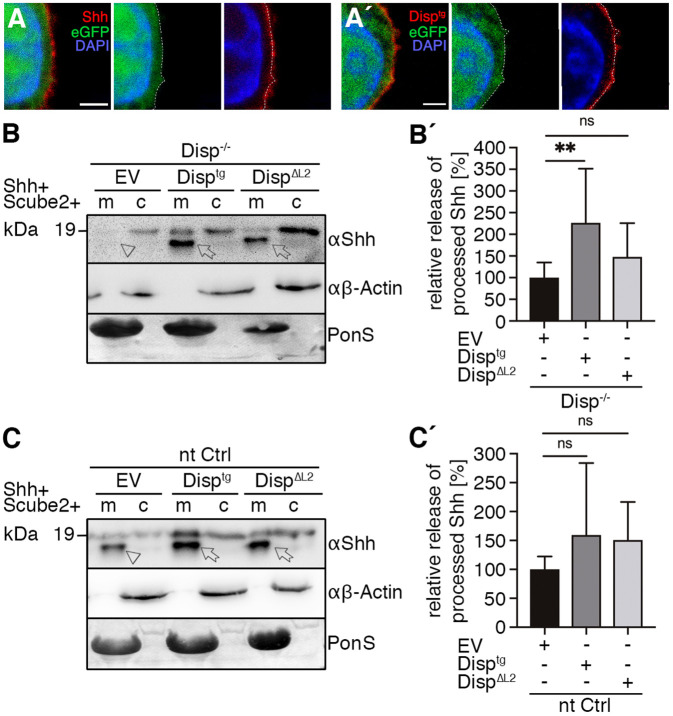
**Overexpressed Disp^tg^ locates to the cell surface and restores Shh release from Disp^−/−^ cells.** (A,A′) Representative confocal planes of Disp^−/−^ cells expressing Shh (A, red) or Disp^tg^ (A′, red). Both transgenes were secreted to the cell surface. Nuclei were counterstained with DAPI (blue). Dashed lines indicate the border of cytoplasmic eGFP signals (green). Images are representative of three experiments. Scale bars: 2 µm. (B) Co-expressed transgenic Disp^tg^ enhanced processed Shh release from Disp^−/−^ cells (c) into the medium (m). Transgenic Disp^ΔL2^, which lacks most of the second extracellular loop, did not release significantly increased amounts of truncated Shh. Empty-vector (EV)-transfected Disp^−/−^ cells served as negative controls. (B′) Quantified relative processed Shh release, as shown in B. Data are mean±s.d. *n*=10. **P<0.01; ns, not significant; *P*=0.377 for Disp^ΔL2^ (one-way ANOVA with Dunnett's multiple comparison post hoc test). (C) Co-expressed transgenic Disp^tg^ or Disp^ΔL2^ did not significantly increase Shh release from nt Ctrl cells. (C′) Quantified relative processed Shh release as shown in C. Data are mean±s.d. *n*=14. ns, not significant (one-way ANOVA with Dunnett's multiple comparison post hoc test). In B and C, arrows indicate solubilized truncated Shh from Disp^tg^- or Disp^ΔL2^-expressing cells and the arrowhead indicates solubilized Shh from EV-transfected cells. Anti-β-actin blots (αβ-actin) and Ponceau S staining of residual serum albumin (PonS) serve as loading controls.

**Fig. 4.**
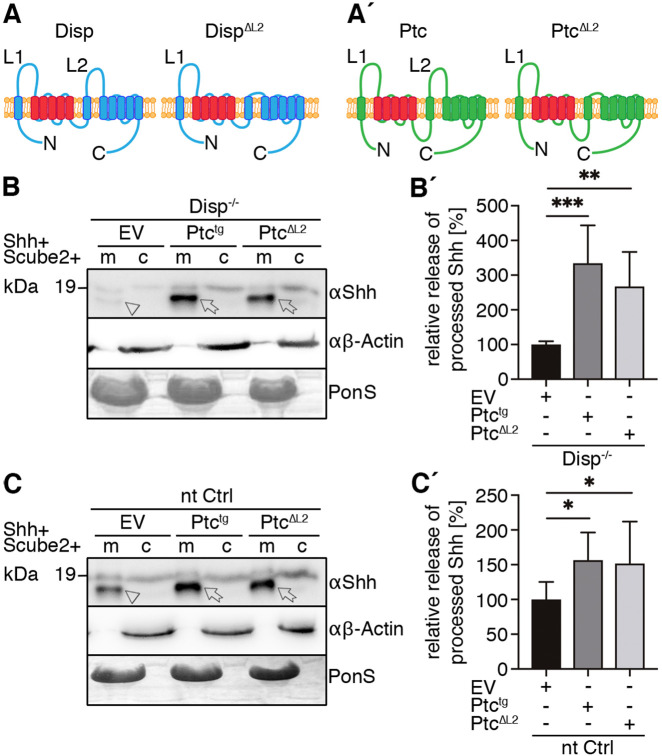
**Overexpressed Ptc^tg^ restores Shh release from Disp^−/−^ cells.** (A,A′) Schematic representations of Disp (blue) and Ptc (green). Twelve transmembrane domains (TM1–TM12), two extracellular loops (L1 and L2), and the N- and C-termini are labeled. Conserved SSDs (TM2–TM6) are highlighted in red. Disp^ΔL2^ and Ptc^ΔL2^ lack most of the second extracellular loops (L2). (B,C) Co-expression of transgenic Ptc^tg^ or Ptc^ΔL2^ increases Shh shedding from Disp^−/−^ (B) and nt Ctrl (C) cells (c, cellular Shh; m, Shh released into the medium). Arrows indicate solubilized processed Shh from Ptc^tg^- or Ptc^ΔL2^-expressing cells, and arrowheads indicate reduced amounts of solubilized Shh from empty vector (EV)-transfected cells. Anti-β-actin blots (αβ-actin) and Ponceau S staining of residual serum albumin (PonS) serve as loading controls. (B′,C′) Quantification of relative processed Shh release as shown in B and C. Data are mean±s.d. *n*=6 in B′, *n*=9 in C′. *P<0.05, **P<0.01, ***P<0.001 (one-way ANOVA with Dunnett's multiple comparison post hoc test).

Next, we reversed the Disp^−/−^ phenotype by the overexpression of transgenic V5-tagged Disp^tg^ (Stewart et al., 2018). Confocal microscopy of non-permeabilized Disp^−/−^ cells expressing either Shh or Disp^tg^ confirmed secretion of both proteins to the cell surface (Fig. 3A,A′). Consistent with their cell-surface localization, co-expressed Disp^tg^ restored Shh shedding from Disp^−/−^ cells (Fig. 3B, arrows, B′). We also tested the activity of a murine Disp^ΔL2^ variant lacking amino acids 752–972 of the second extracellular loop, located between transmembrane (TM) regions 7 and 8 (Fig. 4A), to determine a possible role of this loop in Shh binding and release (Cannac et al., 2020). This assay did not reveal significantly increased Shh shedding from Disp^ΔL2^-expressing Disp^−/−^ cells (Fig. 3B,B′), indicating that the Disp L2 region contributes to the process. Shh shedding from Disp^tg^- and Disp^ΔL2^-transfected nt Ctrl cells was also not significantly increased, indicating sufficient endogenous Disp expression in these cells (Fig. 3C,C′).

How can control of Shh shedding by Disp be explained? One answer to this question comes from established structural similarities between Disp and Ptc: both proteins have 12 TM regions and both contain SSDs (consisting of TM2–TM6; colored red in Fig. 4A,A′) that can be superimposed and accommodate several sterol-like densities in a central hydrophobic conduit (Cannac et al., 2020; Gong et al., 2018). The Ptc SSD also possesses similarities to prokaryotic RND transporters that function as proton-driven antiporters, often to export molecules through a hydrophobic channel that resembles the conduit of Ptc (Nies, 1995; Tseng et al., 1999). Ptc may thus use this conduit to transport cholesterol, and while active Ptc decreases free cellular cholesterol (Bidet et al., 2011), Hh association with its extracellular loops L1 and L2 stops SSD-mediated cholesterol transport. From these findings, we hypothesized that Disp may likewise transport free (unesterified) cellular cholesterol, 60–80% of which resides in the plasma membrane (van Meer et al., 2008). If this is correct, we further hypothesized that co-expression of transgenic murine Ptc (Ptc^tg^) with Shh–Hhat and Scube2, might compensate for Disp loss in our mutant cell line. To test these hypotheses, we co-transfected Disp^−/−^ cells and nt Ctrl cells with Ptc^tg^ or constitutively active murine Ptc that lacks the Shh-binding L2 region (Ptc^ΔL2^; Fig. 4A′), which renders the molecule insensitive to activity downregulation by Shh (Taipale et al., 2002). As expected, Ptc^tg^ and Ptc^ΔL2^ strongly increased Shh shedding from Disp^−/−^ cells (Fig. 4B, arrows, B′), as well as from control cells (Fig. 4C, arrows, C′). This observation suggests that Ptc and Disp can act in similar mechanistic manner to increase the release of truncated Shh, and that this mechanism may depend to a large part on the SSDs that are conserved in both proteins. These, in turn, may act on the amount or distribution of cholesterol in the plasma membrane.

**Fig. 5.**
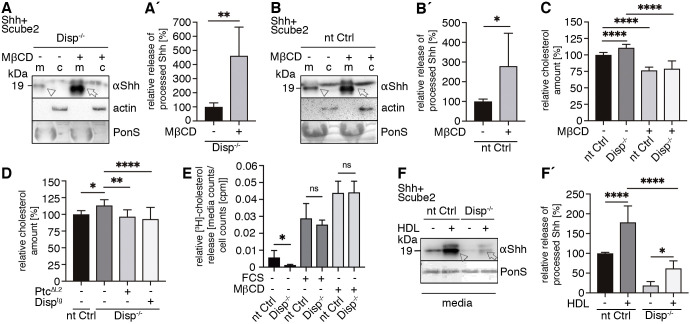
**Disp-deficient cells contain increased membrane cholesterol, and cholesterol depletion increases Shh shedding.** (A–B′) The membrane-cholesterol-depleting drug MβCD enhances Shh shedding from Disp^−/−^ cells (A,A′) and nt Ctrl cells (B,B′). Solubilized Shh in the absence or presence of MβCD is indicated by an arrowhead or an arrow, respectively (c, cellular Shh; m, Shh released into the medium). In A and B, anti-β-actin blots and Ponceau S staining of residual serum albumin (PonS) serve as loading controls. Data in A′ and B′ are mean±s.d. *n*=6 in A′, *n*=5 in B′. *P<0.05, **P<0.01 (two-tailed unpaired *t*-test). (C) Quantified relative free cholesterol content in nt Ctrl and Disp^−/−^ cells in the presence or absence of MβCD. Data are mean±s.d. nt Ctrl and Disp^−/−^, *n*=29; both MβCD-treated groups, *n*=9. ****P<0.0001 (one-way ANOVA with Sidak's multiple comparison post hoc test). (D) Quantified relative free cholesterol content in nt Ctrl cells, Disp^−/−^ cells, and Disp^−/−^ cells expressing Ptc^ΔL2^ or Disp^tg^. Data are mean±s.d. nt Ctrl *n*=14, all Disp^−/−^ groups *n*=13. *P<0.05, **P<0.01, ****P<0.0001 (one-way ANOVA with Sidak's multiple comparison post hoc test). (E) The indicated cell lines were loaded with [^3^H]-cholesterol for 48 h then washed, and cholesterol release into serum-depleted medium (FCS−) or medium with 10% FCS (FCS+) was measured for 3 h. [^3^H]-cholesterol signals in media are expressed relative to cellular counts. MβCD treatment (MβCD+; 1 mg/ml) served as a positive control. FCS− MβCD− *n*=6, FCS+ MβCD− *n*=5, FCS− MβCD+ *n*=3 for each cell line. Data are mean±s.d. *P<0.05; ns, not significant (two-tailed unpaired *t*-test). (F) HDL enhances Shh release into the medium from nt Ctrl cells (arrowhead) and to a lesser degree from Disp^−/−^ cells (arrow). Ponceau S staining of residual serum albumin (PonS) serves as loading control. (F′) Quantification of relative processed Shh release, as shown in F. Data are mean±s.d. nt Ctrl groups *n*=6, Disp^−/−^ groups *n*=5. *P<0.05, ****P<0.0001 (one-way ANOVA with Sidak's multiple comparison post hoc test).

To test this idea, and to test whether Disp function is related to the postulated cholesterol transporter-like activity of Ptc, we depleted Disp^−/−^ cells of free membrane cholesterol using the cholesterol-extracting drug methyl-β-cyclodextrin (MβCD) (Zidovetzki and Levitan, 2007). Indeed, MβCD treatment restored the release of bioactive (Fig. S2C) Shh from Disp^−/−^ cells in a concentration-dependent manner (Fig. 5A, arrow, A′; Fig. S3A,C) and shedding was also increased in MβCD-treated nt Ctrl cells (Fig. 5B, arrow, B′; Fig. S3B,D). Of note, the possibility that MβCD extracts lipidated Shh was ruled out by the observation that all released Shh was processed, which should not be the case if Shh extraction from the membrane had occurred. Our findings therefore show that pharmacological membrane cholesterol depletion increases proteolytic Shh processing and support that Ptc and Disp may regulate free membrane cholesterol content in a similar manner. To further investigate this possibility, we quantified total (esterified) and free cholesterol in Disp^−/−^ and nt Ctrl cells (Fig. 5C; Fig. S4A–G). In line with an established function as a membrane cholesterol extractor (Zidovetzki and Levitan, 2007), the pharmacological MβCD control significantly reduced the amounts of free cellular cholesterol in our assay (Fig. 5C; Fig. S4F,G). We hypothesized that, if Disp also extracts membrane cholesterol, we should observe an increase in free cholesterol in Disp^−/−^ cells compared with the levels in nt Ctrl cells. Our quantitative assay supported this hypothesis, because free cholesterol amounts in Disp^−/−^ cells were significantly increased over those in nt Ctrl cells (Fig. 5C; Fig. S4), and because Disp^tg^ and Ptc^ΔL2^ expression in Disp^−/−^ cells reduced free cholesterol amounts in the same assay (Fig. 5D). This suggests that Ptc and Disp can both deplete free cholesterol from the plasma membrane, possibly via their SSDs. To test this possibility, we directly quantified cholesterol efflux from Disp^−/−^ cells and nt Ctrl cells using tritiated cholesterol. Notably, we determined significantly reduced [^3^H]-cholesterol egress into serum-depleted supernatants of Disp^−/−^ cells and also reduced [^3^H]-cholesterol release into supernatants of Disp^−/−^ cells supplemented with 10% fetal calf serum (FCS), despite the expression of ATP-binding cassette (ABC) transporters A1 and G1 that also export cellular sterols (Fig. 5E). Consistent with the quantitative assay results described above (Fig. 5C,D), we found that cellular [^3^H]-cholesterol counts in Disp^−/−^ cell lysates were increased over those in nt Ctrl cells [nt Ctrl, 83,840±4682 counts per minute (cpm), *n*=4; Disp^−/−^, 106,700±12,097 cpm, *n*=4; mean±s.d. *P*=0.0123, two-tailed unpaired *t*-test]. As a positive pharmacological control, MβCD extracted similar amounts of [^3^H]-cholesterol from nt Ctrl cells and Disp^−/−^ cells (Fig. 5E). Based on these findings, we suggest that, like Ptc (Zhang et al., 2018), Disp depletes free cholesterol from the plasma membrane. We also suggest that the extracted cholesterol is then transferred to a sink – possibly a soluble carrier – and subsequently transported away from the cell. In invertebrates, known soluble cholesterol sinks include lipoproteins called lipophorins. In vertebrates, high-density lipoproteins (HDLs) are a specialized lipoprotein fraction that accepts peripheral cholesterol from transmembrane transporters of the ABC family (Luo et al., 2020). This function would make HDLs suited to accepting the sterol-like densities present in the conserved SSDs of Ptc and Disp (Cannac et al., 2020; Zhang et al., 2018). To test this potential, we added purified human HDL to serum-free medium of Shh-expressing Disp^−/−^ cells and nt Ctrl cells. We expected that added HDL would increase proteolytic Shh processing from nt Ctrl cells and that Disp^−/−^ cells would be less responsive due to the lack of the Shh-specific Disp exporter. Indeed, HDL significantly increased the release of processed Shh from nt Ctrl cells (Fig. 5F, arrowhead, F′; Fig. S5A,C) and to a much lesser degree from Disp^−/−^ cells (Fig. 5F, arrow, F′; Fig. S5B,C). Therefore, our results suggest that established extracellular cholesterol carriers such as HDL may act downstream of Disp to accept free membrane sterol. This, in turn, may decrease local cholesterol concentration or distribution at the cell surface to stimulate Shh shedding, as seen as a consequence of pharmacological cholesterol depletion by MβCD.

## DISCUSSION

Although Disp is firmly established as a critical component of the Hh pathway, two outstanding questions have remained unanswered: (1) What is the molecular mechanism by which Disp releases lipid-modified Shh from producing cell membranes? (2) Are there additional Disp functional partners to promote Shh deployment? In this study, we provide answers to these questions and suggest a model for Hh release that matches several predictions derived from previous genetic studies (Burke et al., 1999; Kawakami et al., 2002; Ma et al., 2002). Our model of Disp-modulated Shh shedding is consistent with the prediction that Disp is only required in Hh-producing cells and not in receiving cells. It also accords with the prediction that Hh signaling defects in Disp-deficient model organisms are not due to a defect in Hh production or cholesteroylation, but rather to the deployment of lipidated (but not artificially produced unlipidated forms of) Shh from producing cells. Furthermore, our data support the prediction that full Disp activity in Shh release requires Scube2 (Tukachinsky et al., 2012) and can further explain why the Disp loss-of-function phenotype in *Drosophila* is less severe than that of the single Hh ortholog (Burke et al., 1999). We suggest that, although Disp is required for regulated Hh shedding, it is not absolutely essential, as has previously been shown for some Hh-producing tissue types *in vivo* (Nakano et al., 2004; Tsiairis and McMahon, 2008) and as we now show for transfected cells that overexpress Shh *in vitro*. Finally, we directly demonstrate that Disp increases membrane cholesterol export and that established lipid carriers such as HDL may act as cholesterol acceptors, at least in our *in vitro* system. These findings suggest that Disp extracts cholesterol, or that it may contribute to a heteroprotein membrane complex that extracts the sterol from the plasma membrane as a prerequisite for its removal from the cell. In this regard, Disp might act like bacterial RND transporters and the related Niemann–Pick Type C protein 1 (NPC1) and Ptc (both are SSD-containing transmembrane proteins that pump cholesterol). If correct, our model, which suggests that Disp can export cholesterol and that lipoproteins can act as cholesterol sinks, would provide an explanation for the published observations that knockdown of fly lipophorin impairs Hh biofunction *in vivo* (Panakova et al., 2005), that cytonemes that transport Hh to target cells contain Disp at their tips (reviewed in Hall et al., 2019), and that Ptc acts as a lipoprotein receptor *in vivo* (Callejo et al., 2008). Although our study did not directly address lipoprotein function in the fly, the possibility exists that Disp-mediated cholesterol relay to these carriers supports proteolytic processing of terminal lipidated Hh peptides, and enhances *in vivo* signaling by the truncated protein (Palm et al., 2013), for example at the tips of cytonemes. This model indicates that free plasma membrane cholesterol not only stands out as one endogenous ligand of Ptc to regulate the Hh pathway in receiving cells (Zhang et al., 2018), but that it can also act as a physiological second messenger in Shh-producing cells. This possibility is supported by structural similarities between Ptc and Disp, including shared TM topology and the presence of very similar SSDs in both proteins (Cannac et al., 2020).

We note that our results contrast with recent reports of Disp-mediated extraction of apparently full-length (unprocessed) Shh under similar experimental conditions (0–2% FCS; summarized in Hall et al., 2019). These differences can be explained by the use of our unique Shh–Hhat co-expression system to generate dual-lipidated membrane-associated Shh in a near-complete manner (Jakobs et al., 2014). Only such dual-lipidated proteins reliably undergo Scube2-modulated proteolytic processing at both terminal peptides during release to generate truncated delipidated Shh, as clearly indicated by an electrophoretic size shift and reduced hydrophobicity (Fig. 1). In contrast, Shh expression under similar experimental conditions but in the absence of exogenous Hhat, as conducted by others (Creanga et al., 2012; Tukachinsky et al., 2012; Wierbowski et al., 2020), might result in insufficient N-palmitoylation and membrane-association of the N-terminal peptide, which in turn may lead to an alternative release of N-terminally unprocessed morphogens (Jakobs et al., 2014). Finally, we note that a dispensable role for one or both Hh lipids in eliciting signaling at receiving cells, as implicated by our hypothesis of Disp- and Scube2-modulated Shh shedding, is supported by recent evidence of Ptc activity regulation as a consequence of (unlipidated) nanobody binding to its extracellular loop L1 (Zhang et al., 2020), the exact site that represents the physiological Shh–Ptc protein-binding interface (Gong et al., 2018).

## MATERIALS AND METHODS

### Cell lines

Bosc23 cells and C3H10T1/2 reporter cells were cultured in DMEM (PAN Biotech, Aidenbach, Germany) supplemented with 10% FCS (PAN Biotech) and 100 µg/ml penicillin-streptomycin (PAN Biotech).

### Generation of Disp^−/−^ cells using CRISPR/Cas9

Disp1-knockout cells (Disp^−/−^) were generated from Bosc23 cells according to the manufacturer's protocol (Dharmacon). The following RNAs and plasmids were used: (1) Edit-R Human DISP1 crRNA (CM-013596-02-0002), (2) Edit-R CRISPR-Cas9 Synthetic tracrRNA (U-002005-20), (3) Edit-R hCMV-PuroR-Cas9 Expression Plasmid (U-005100-120), and (4) Edit-R crRNA Non-targeting Control #1 (U-007501-01-05). Disp1 knockout was confirmed via sequencing of PCR products generated from the CRISPR-Cas9 targeted Disp DNA target site followed by the sequencing of ten individual (cloned) PCR products. This strategy revealed three Disp1 loci in Bosc23 cells, consistent with the parental HEK293 cell line modal chromosome number of 64 (https://www.phe-culturecollections.org.uk/products/celllines/). Complete Disp1 knockout was confirmed by immunoblotting with anti-Disp1 antibodies (1:1000; R&D Systems, AF3549). Non-targeting control (nt Ctrl) guide RNA did not change Disp DNA sequence or protein expression in control cells. Off-targets predicted by CRISPOR (http://crispor.tefor.net/) were analyzed by DNA sequencing, and their wild-type sequence was confirmed (Table S1). Independently generated nt Ctrl and Disp^−/−^ cell lines were used to confirm impaired Shh release and cholesterol quantification (shown in Fig. S5B). In all assays, independently generated nt Ctrl and Disp^−/−^ cell lines behaved like the lines presented in this work.

### Cloning of recombinant proteins

Shh expression constructs were generated from murine cDNA (NM_009170; nucleotides 1–1314, corresponding to amino acids 1–438; for ShhN, nucleotides 1–594 were used, corresponding to amino acids 1–198) and human hedgehog acyltransferase (Hhat) cDNA (NM_018194). Both cDNAs were cloned into pIRES (Clontech) for their coupled expression from bicistronic mRNA to achieve near-quantitative Shh palmitoylation. Unlipidated ^C25S^ShhN cDNA and non-palmitoylated ^C25A^Shh cDNA (amino acids 1–438, the alanine modification completely blocks Shh N-palmitoylation, while the serine modification reduces it; Hardy and Resh, 2012) were generated by site-directed mutagenesis (Stratagene) and inserted into pcDNA3.1 (Invitrogen). Primer sequences are provided in Table S3. Human Scube2 constructs were a kind gift from Ruey-Bing Yang (Academia Sinica, Taiwan). Murine V5- and C-terminal HA-tagged Disp^tg^ inserted into pcDNA3.1 was a kind gift from Stacey Ogden (St. Jude Children's Research Hospital, Memphis, USA). Murine Disp^ΔL2^ was generated from Disp^tg^ by deletion of the second extracellular loop (L2) between TM domain 7 and 8 (amino acids 752–972). Murine Ptc^ΔL2^ was generated from Ptc full length (pcDNA-h-mmPtch1-FL; Addgene 120889) by deletion of the second extracellular loop (L2) between TM domain 7 and 8 (amino acids 794–997).

### Protein detection

Bosc23 cells were seeded into 6-well plates and transfected with 0.5 µg Disp- or Ptc-encoding constructs and 0.5 µg Shh constructs (for Disp or Ptc co-transfection experiments) or 1 µg Shh constructs with or without 0.5 µg Scube2 construct per well using Polyfect (Qiagen). Cells were grown for 2 d, or 3 d for Disp^−/−^ rescue experiments, at 37°C with 5% CO_2_ in DMEM containing 10% FCS and penicillin-streptomycin (100 µg/ml). The medium was changed to serum-free DMEM for 6 h, before cells were harvested and centrifuged at 300 ***g*** for 10 min to remove debris. Supernatants were incubated with 10% trichloroacetic acid (TCA) for 30 min on ice, followed by centrifugation at 13,000 ***g*** for 20 min to precipitate the proteins. Cell lysates (in 300 µl reducing SDS–PAGE buffer, 15 µl applied to the gel) and corresponding supernatants (in 15 µl reducing SDS–PAGE buffer) were analyzed on the same reducing SDS-polyacrylamide gel and detected by western blot analysis using goat anti-Shh antibodies (1:2000; R&D Systems, AF464), mouse anti-β-actin antibodies (1:10,000; Sigma-Aldrich, A3854) or mouse anti-HA antibodies (1:1000; Sigma-Aldrich, H9658) followed by incubation with horseradish peroxidase-conjugated secondary antibodies. β-actin (for cell lysates) and Ponceau S (for media) served as loading controls. Note that due to their different dilutions (15 µl reducing SDS–PAGE buffer to dissolve the TCA-precipitated released material, 300 µl reducing SDS–PAGE buffer to dissolve the cellular material), positive or negative changes in Shh release on western blots do not correlate 1:1 with negative or positive changes in the cellular signals. Shh release was quantified using ImageJ (NIH, Bethesda, MD, USA) and calculated as the ratio of total or processed (truncated) soluble Shh relative to the cellular Shh material. Relative Shh release from control cells (nt Ctrl) was then set to 100%, and Shh release from Disp^−/−^ cells was expressed relative to that value. For rescue experiments, Shh release from empty vector-transfected control cells was set to 100%. In cholesterol-depletion experiments, serum-free medium was supplemented with 0–800 µg/ml methyl-β-cyclodextrin (MβCD) for 6 h prior to TCA precipitation and subsequent immunoblotting analysis, and Shh release from mock-treated cells was set to 100%.

### Shh release in the presence of high-density lipoprotein

nt Ctrl or Disp^−/−^ cells were transfected with pIRES for coupled Shh and Hhat expression together with Scube2 cDNA as described above. At 2 d after transfection, cells were washed twice with serum-free DMEM and additionally incubated for 1 h in serum-free DMEM. This extensive washing was intended to completely remove serum lipoproteins. Serum-free DMEM was then discarded, and cells were incubated in serum-free DMEM containing 0–120 µg/ml human HDL (Sigma-Aldrich, L1567) for 6 h. For cell debris removal, supernatants were centrifuged for 10 min at 300 ***g***. For subsequent Shh purification, supernatants were incubated with 5 µg/ml anti-Shh antibody (DSHB, 5E1) for 2 h at 4°C, followed by the addition of 5 mg Protein A–agarose beads (Sigma, P1406) in phosphate-buffered saline (PBS) and incubated at 4°C overnight. Immunoprecipitates were collected by centrifugation at 300 ***g*** for 5 min and subjected to reducing SDS–PAGE followed by immunoblot analysis. Shh release was quantified by determining the ratios of soluble Shh signals detected in 5E1–Protein A pulldown samples relative to cellular actin signals. Shh release from mock-treated nt Ctrl cells (no HDL) was set to 100%.

### Shh bioactivity assay

Bosc23 cells, nt Ctrl, or Disp^−/−^ cells were transfected with Shh–Hhat, or its variants thereof, together with Scube2, as described above. After 2 d, the medium was replaced with serum-free medium for 6 h. Media were then harvested, cellular debris was removed and FCS was added at 10%. Samples were then mixed 1:1 with DMEM supplemented with 10% FCS and 100 μg/ml antibiotics, and the mixture was added to C3H10T1/2 cells. Cells were lysed 6 d after osteoblast differentiation was induced, in 1% Triton X-100 in PBS, and osteoblast-specific alkaline phosphatase activity was measured at 405 nm using 120 mM p-nitrophenyl phosphate (Sigma) in 0.1 M Tris-HCl buffer (pH 8.5). Mock-treated C3H10T1/2 cells served as negative controls.

### Total and free cholesterol quantification

To quantify the content of total (esterified and unesterified) and free (unesterified) cholesterol in nt Ctrl cells and Disp^−/−^ cells, we seeded cells into 6-well plates and grew the cells in DMEM containing 10% FCS and 100 μg/ml penicillin-streptomycin at 37°C for 2 d. For experiments containing MβCD, cells were incubated with 800 µg/ml MβCD for 6 h in serum-free medium prior to cell lysis. For expression of Ptc^ΔL2^ and Disp^tg^ in Disp^−/−^ cells, cDNAs (1 µg/well of one 6-well plate) were transfected as described above, and cells were grown for 3 d prior to cell lysis. Afterwards, cells were washed twice with PBS, harvested using cell scrapers in 1 ml PBS and centrifuged at 800 ***g*** for 5 min at 4°C. The resulting cell pellets resuspended in 500 µl HB buffer (250 mM sucrose, 3 mM imidazole at pH 7.4) for washing. Samples were centrifuged again, and cell pellets were resuspended in 400 µl HB buffer. Cells were subsequently mechanically lysed using a syringe (27G), and nuclei were removed by centrifugation at 1000 ***g*** for 15 min at 4°C. Total protein concentration of supernatants were measured at 280 nm using a NanoDrop spectrophotometer and adjusted to similar levels. For total and free cholesterol quantification, an Amplex Red cholesterol assay (Invitrogen, A12216) was conducted according to the manufacturer's protocol with or without the use of the cholesterol esterase (converts esterified cholesteryl into free cholesterol), respectively.

### Cholesterol efflux assay

To conduct this assay, we followed a published protocol (Low et al., 2012). Briefly, Disp^−/−^ cells and nt Ctrl cells were seeded in 12-well plates at a final density of 0.2×10^6^ cells per well in 0.9 ml DMEM containing 10% FCS and 100 μg/ml penicillin-streptomycin, and cells were incubated at 37°C, 5% CO_2_. After 24 h, the medium was changed for DMEM containing 10% FCS, 100 μg/ml penicillin-streptomycin and 0.5 µCi [^3^H]-cholesterol (Perkin-Elmer, Foster City, USA) per well. After 2 d, the medium containing the [^3^H]-cholesterol was removed, the cells were gently washed, and serum-free medium with 0.1% BSA was added. After 18 h, cells were checked under the microscope for confluency, and the medium was exchanged for 250 µl serum-free medium or medium containing 1 mg/ml MβCD or medium containing 10% FCS. After 3 h, cells and medium were harvested and transferred into scintillation vials, [^3^H] signals were counted, and the amount of released [^3^H]-cholesterol was expressed as the proportion of solubilized [^3^H]-cholesterol detected in the medium (minus the blank efflux) divided by the cellular [^3^H]-cholesterol amounts after normalization for protein content.

### Confocal microscopy

Disp^−/−^ cells were seeded onto gelatin-coated 4-well cell culture chamber slides (PAA, PAA30104X) and transfected with either V5-tagged Disp^tg^ or Shh together with Hhat and Scube2 using Polyfect, as described above. 0.5 μg pEGFPN1 (Clontech) was co-transfected to visualize the cytoplasm of the cell. After 2 d in culture, cells were fixed with 4% paraformaldehyde (PFA) for 10 min at room temperature under non-permeabilizing conditions. Mouse anti-Shh (1:170, 5 μg/ml; DSHB, 5E1) and mouse anti-V5 antibodies (1:100; Abcam, ab27671) were used to stain Shh and Disp, respectively. Texas Red-conjugated goat anti-mouse IgG antibodies (1:300; BioRad Serotech, Feldkirchen, Germany; 107007) and a Zeiss LSM700 confocal microscope were used for visualization. DAPI was used as a nuclear counterstain. Fiji was used to merge the fluorescence channels, and a representative single slice is shown.

### Reverse-phase high-performance liquid chromatography

Bosc23 cells were transfected with expression plasmids for unlipidated ^C25A^ShhN control protein and cholesteroylated (yet non-palmitoylated) ^C25A^Shh. At 2 d after transfection, cells were lysed in radioimmunoprecipitation assay buffer containing Complete protease inhibitor cocktail (Roche, Basel, Switzerland) on ice and ultracentrifuged, and the soluble whole-cell extract was acetone precipitated. Protein precipitates were resuspended in 35 µl of (1,1,1,3,3,3) hexafluoro-2-propanol and solubilized with 70 µl of 70% formic acid, followed by sonication. Reverse-phase HPLC was performed on a C4-300 column (Tosoh, Tokyo, Japan) and an Äkta Basic P900 Protein Purifier. To elute the samples, we used a 0–70% acetonitrile/water gradient with 0.1% trifluoroacetic acid at room temperature for 30 min. Eluted samples were vacuum dried, resolubilized in reducing sample buffer, and analyzed by SDS–PAGE and immunoblotting using anti-Shh antibodies (1:2000; R&D Systems, AF464). Signals were quantified using ImageJ and normalized to the highest protein amount detected in each run.

### Bioanalytical and statistical analysis

All statistical analyses were performed in GraphPad Prism. Applied statistical tests, post hoc tests and number of independently performed experiments are stated in the figure legends. A *P*-value of <0.05 was considered statistically significant. *P<0.05, **P<0.01, ***P<0.001 and ****P<0.0001 in all assays. Error bars represent the s.d. of the mean. The s.d. shown for Shh protein expression and release from nt Ctrl cells on western blots represents variation from the average value (set to 100%) detected on the same blot. Mean±s.d. values for data shown in the figures are provided in Table S2.

## Supplementary Material

Supplementary information

Reviewer comments
