# Supplementary material for: Conserved cholesterol-related activities of Dispatched 1 drive Sonic hedgehog shedding from the cell membrane
Source: J Cell Sci. 2021 Aug 19;135(5):jcs258672. doi: 10.1242/jcs.258672 (PMC8403983; doi:10.1242/jcs.258672)
Supplement: Supplementary information [file joces-135-258672-s1.pdf]

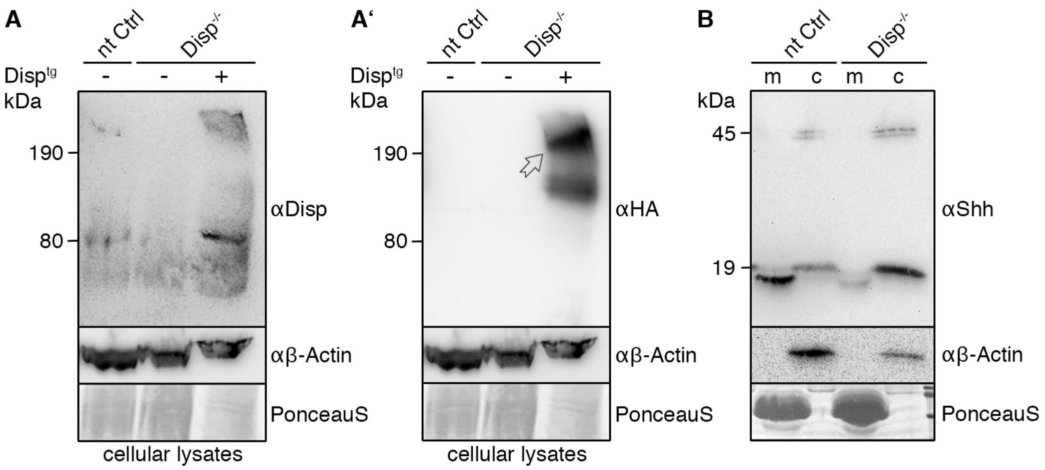

Fig. S1. Loss of Disp protein expression and unimpaired Shh autoprocessing in Disp$^{-/-}$ cells. **A)** Disp$^{-/-}$ cellular lysates were analyzed by SDS-PAGE/immunoblotting. Full-length Disp (exceeding 190kDa) and degradation products (80kDa) were detected in nt Ctrl and transfected (Disp^{tg}-expressing) Disp$^{-/-}$ cells. Untransfected Disp$^{-/-}$ cells lack both signals (center lane). **A´)** Notably, Disp clusters do not fully disrupt but partially degrade under SDS-PAGE conditions (Stewart et al., 2018), which is confirmed on the same (stripped) blot by specific detection of HA-tagged Disp^{tg} at the established (Stewart et al., 2018) increased size (arrow). **B)** Similar autoprocessing of 45kDa Shh precursor proteins into cholesteroylated, 19kDa products in nt Ctrl and Disp$^{-/-}$ cells (c). Note that media (m) contain soluble Shh proteins that show increased electrophoretic mobility over their cellular precursors (c).

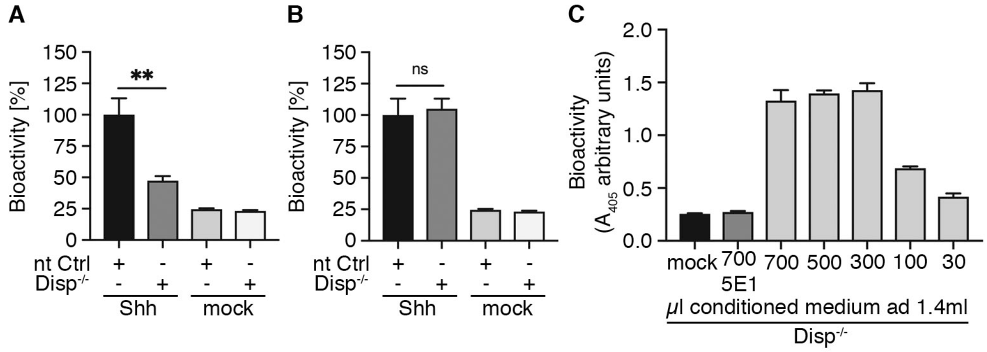

Fig. S2. Soluble truncated Shh and MβCD-released Shh are bioactive. **A)** Quantification of Shh bioactivity. Shh was released from transfected nt Ctrl and Disp$^{-/-}$ cells, the conditioned medium adjusted to 10% FCS, added to C3H10T1/2 reporter cells, and the cells incubated for 6 days before analysis. Truncated soluble Shh induced C3H10T1/2 differentiation into alkaline phosphatase-producing osteoblasts. Alkaline phosphatase activity was used as a read-out for Shh biofunction and is expressed relative to C3H10T1/2 cells incubated with supernatants of Shh-transfected nt Ctrl cells (set to 100%). Compared with Shh release from nt Ctrl cells, reduced Shh solubilization from Disp$^{-/-}$ cells corresponded well with reduced Shh bioactivity. n=3, unpaired two-tailed t-test. **B)** After protein normalization, Shh released from Disp$^{-/-}$ cells was as bioactive as the material released from nt Ctrl cells. This finding supports that reduced Shh biofunction as a consequence of Disp deletion solely results from reduced Shh solubilization and not from the possible secretion of "inactivated" proteins. It also demonstrates that the associated loss of the N-palmitate does not abolish Shh biofunction. n=3, unpaired two-tailed t-test. **C)** Shh was released over night from transfected Disp-/- cells in the presence of 500µg/ml MbCD, indicated amounts of supernatant adjusted to 1.4ml and added to C3H10T1/2 cells. Alkaline phosphatase activity was directly determined as a read-out for Shh bioactivity. 1.5µg 5E1 (an antibody established to interfere with Shh/Ptc receptor binding) completely blocked Shh biofunction, confirming specificity of the assay. n=3 datasets from 1 experiment.

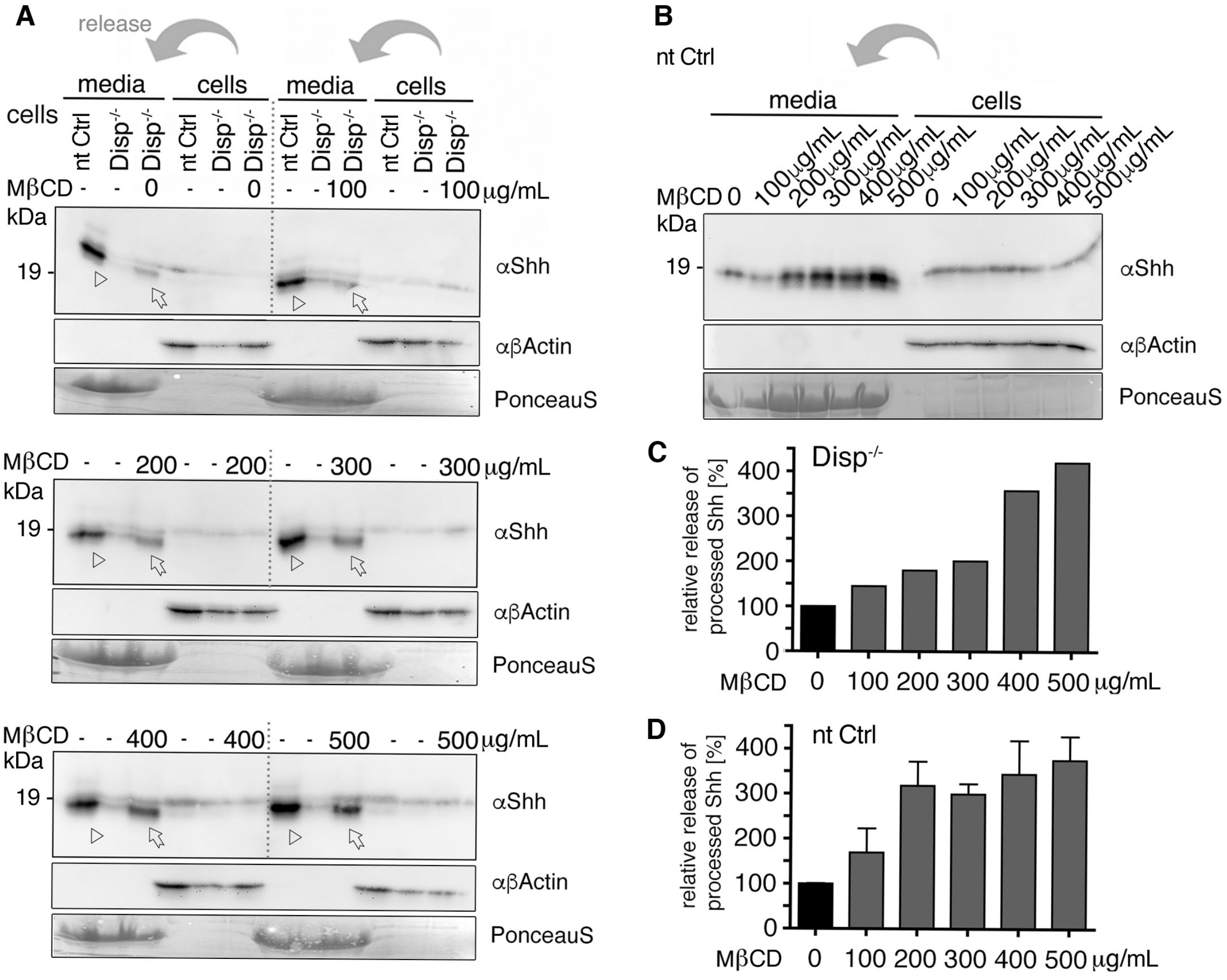

Fig. S3. MβCD increases Shh release from nt Ctrl and from Disp$^{-/-}$ cells in dose-dependent manner. A) 0 to 500 µg/mL MβCD increased processed Shh release from Disp$^{-/-}$ cells over a 6 h period (arrows). Shh release was compared with untreated Disp$^{-/-}$ cells (adjacent lanes on the left) and Shh amounts released from nt Ctrl cells in the absence of MβCD (far left lanes, arrowheads). Anti-β-Actin and PonceauS served as loading controls. **B)** 0 to 500 µg/mL MβCD were used to release processed Shh from nt Ctrl cells. **C, D)** Quantification of processed Shh release as shown in A, B.

Journal of Cell Science • Supplementary information

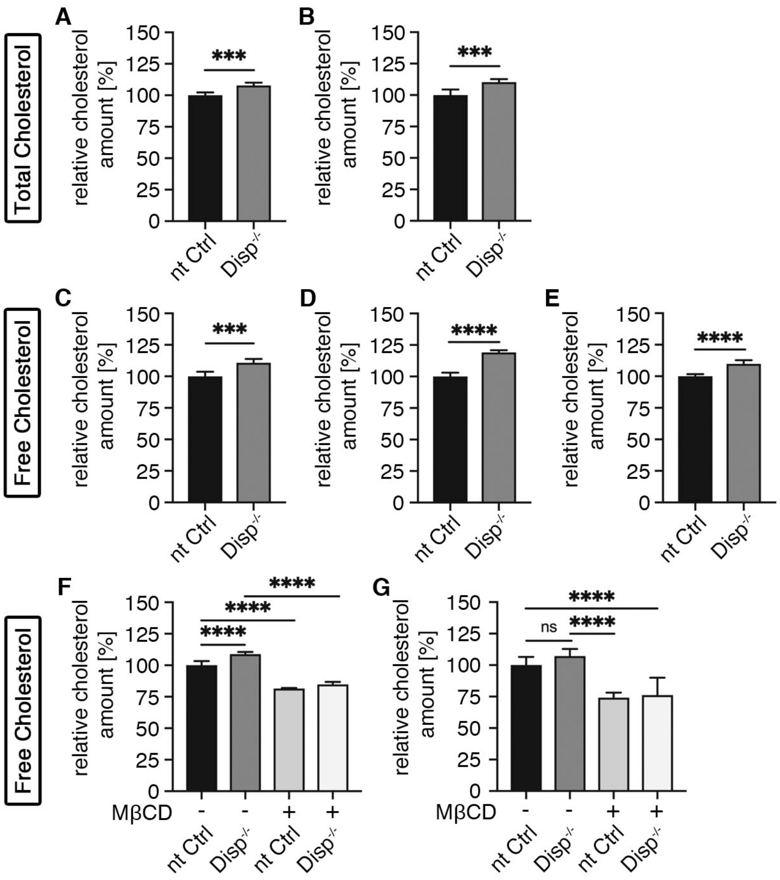

Fig. S4. Quantification of total and free (unesterified) cholesterol in nt Ctrl and Disp$^{-/-}$ cells. Two out of two conducted total cholesterol quantification assays (**A, B**) and four of five conducted free cholesterol quantification assays (**C-F**) revealed increased cholesterol in Disp$^{-/-}$ cells if compared with nt Ctrl cells. Only one out of seven independently conducted assays (biological replicates) did not demonstrate a significant cholesterol increase in Disp$^{-/-}$ cells (G). Pooled results from C-G are shown in Fig. 4C. MβCD consistently depleted free cellular cholesterol from the cells (**F, G**), confirming validity of the assay. A: n=5. B: A second independent nt Ctrl and Disp$^{-/-}$ cell line displayed similar results, n=6. C: n=6. D: n=5. E: n=6. F: n=6. G: n=6. A, B, C, D, E: Unpaired two-tailed t-test in each assay. F, G: One-way ANOVA, Sidak's multiple comparison post hoc test in each assay.

Journal of Cell Science • Supplementary information

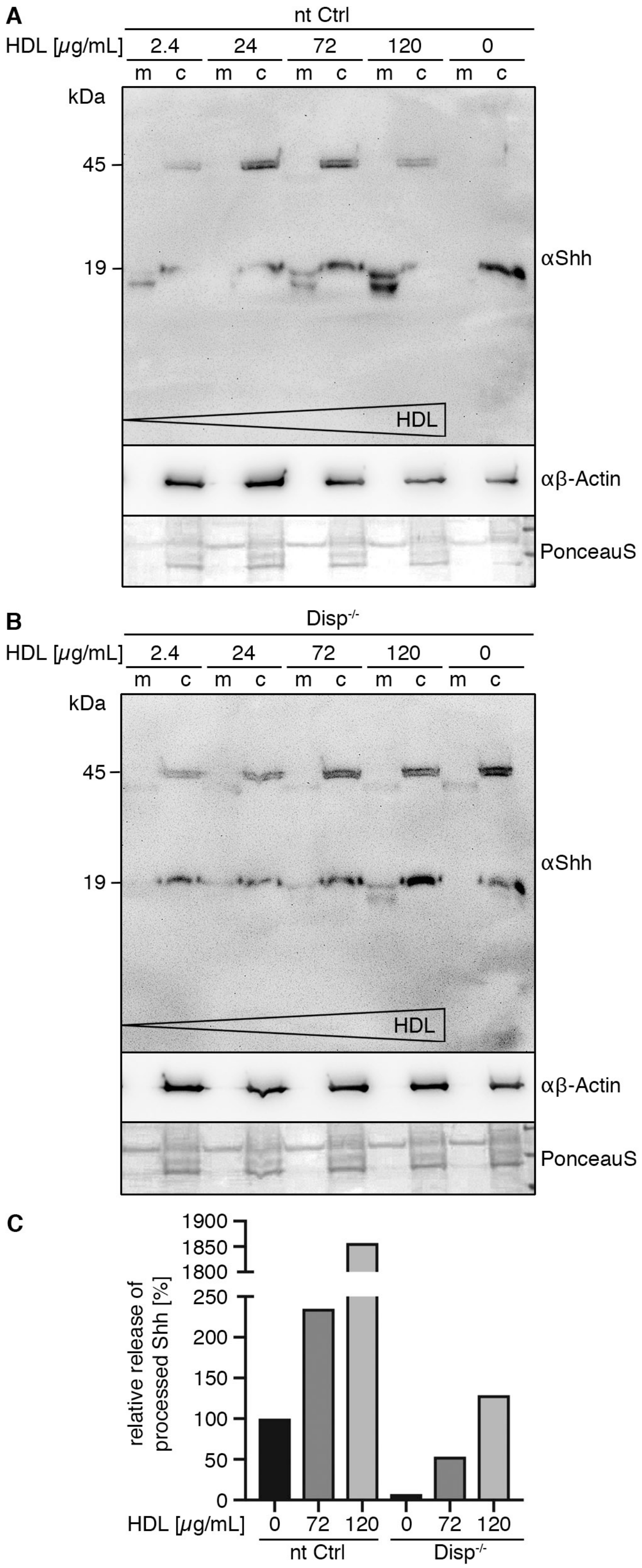

Fig. S5. HDL dose-dependently enhances Shh release in nt Ctrl cells, but much less so in Disp$^{-/-}$ cells. A, B) HDL added to serum free media, ranging in concentrations from 0 µg/mL to 120 µg/mL, increasingly enhanced Shh release in nt Ctrl cells but much less so from Disp$^{-/-}$ cells. Anti-β-Actin and PonceauS served as loading controls. **C)** Quantification of the relative release of processed Shh from lipidated precursors on expressing cells, as shown in A and B.

Journal of Cell Science • Supplementary information

Table S1.

Predicted guide RNA off-targets remained unaffected. Predicted off-target sites of Disp1 guide RNA by CRISPOR were analyzed by DNA sequencing and their wild-type (WT) sequences were confirmed.

Predicted off-target	Accession number	Result
YLPM1	NM_019589	WT sequence confirmed
LINC01304	NR_037881	WT sequence confirmed
APLF	NM_173545	WT sequence confirmed
MINK1	NG_028005	WT sequence confirmed
SLC26A9	NM_052934	WT sequence confirmed

Journal of Cell Science • Supplementary information

Table S2. Raw data used for the figures in this study.

Figure			Value (Mean)	SD
2B′	nt Ctrl		100%	21.07
	$Disp^{-/-}$		26.78%	20.63
2C′	nt Ctrl		100%	29.41
	$Disp^{-/-}$		76.32%	57.46
2D′	nt Ctrl		100%	8.443
	$Disp^{-/-}$		72.66%	30.74
3B′	$Disp^{-/-}$	EV	100%	35.01
	$Disp^{-/-}$	$Disp^{tg}$	226.5%	125.0
	$Disp^{-/-}$	$Disp^{\Delta L2}$	147.9%	77.73
3C′	nt Ctrl	EV	100%	22.17
	nt Ctrl	$Disp^{tg}$	159.3%	124.7
	nt Ctrl	$Disp^{\Delta L2}$	150.7%	65.78
4B′	$Disp^{-/-}$	EV	100%	9.487
	$Disp^{-/-}$	Ptc^{tg}	334.3%	109.0
	$Disp^{-/-}$	$Ptc^{\Delta L2}$	267.4%	99.58
4C′	nt Ctrl	EV	100%	25.27
	nt Ctrl	Ptc^{tg}	156.5%	39.84
	nt Ctrl	$Ptc^{\Delta L2}$	151.8%	60.22
5A′	$Disp^{-/-}$	- MβCD	100%	28.13
	$Disp^{-/-}$	+ MβCD (0.8mg/mL)	462.3%	203.0
5B′	nt Ctrl	- MβCD	100%	11.81
	nt Ctrl	+ MβCD (0.8mg/mL)	279.2%	166.8
5C	nt Ctrl	- MβCD	100%	3.704
	$Disp^{-/-}$	- MβCD	110.8%	5.136
	nt Ctrl	+ MβCD (0.8mg/mL)	76.54%	4.950
	$Disp^{-/-}$	+ MβCD (0.8mg/mL)	79.03%	11.80
5D	nt Ctrl	EV	100%	5.54
	$Disp^{-/-}$	EV	113.4%	8.6
	$Disp^{-/-}$	$Ptc^{\Delta L2}$	96.56%	10.27
	$Disp^{-/-}$	$Disp^{tg}$	92.88%	17.52
5E	nt Ctrl		0.0057	0.0041
	$Disp^{-/-}$		0.0012	0.0004
	nt Ctrl	+ FCS (10%)	0.0289	0.0087
	$Disp^{-/-}$	+ FCS (10%)	0.0251	0.0027
	nt Ctrl	+ MβCD (1mg/mL)	0.0439	0.0069
	$Disp^{-/-}$	+ MβCD (1mg/mL)	0.0442	0.0066
5F′	nt Ctrl	- HDL	100%	2.530
	nt Ctrl	+ HDL (120 µg/mL)	178.3%	41.49
	$Disp^{-/-}$	- HDL	18.80%	9.783
	$Disp^{-/-}$	+ HDL (120 µg/mL)	62.00%	19.01
S2A	nt Ctrl	Shh	100%	13.05
	$Disp^{-/-}$	Shh	47.32%	3.666
	nt Ctrl	untransfected	24.61%	0.778
	$Disp^{-/-}$	untransfected	23.22%	0.696
S2B	nt Ctrl	Shh	100%	13.05
	$Disp^{-/-}$	Shh	105%	8.136
	nt Ctrl	untransfected	24.61%	0.778
	$Disp^{-/-}$	untransfected	23.22%	0.696

Journal of Cell Science • Supplementary information

S2C	$Disp^{-/-}$	mock	0.2550	0.019
	$Disp^{-/-}$	700 5E1	0.2728	0.017
	$Disp^{-/-}$	700	1.328	0.201
	$Disp^{-/-}$	500	1.395	0.059
	$Disp^{-/-}$	300	1.428	0.128
	$Disp^{-/-}$	100	0.6875	0.034
	$Disp^{-/-}$	30	0.4178	0.061
S3C	$Disp^{-/-}$	0 (MβCD µg/mL)	100%	
	$Disp^{-/-}$	100 (MβCD µg/mL)	145%	
	$Disp^{-/-}$	200 (MβCD µg/mL)	180%	
	$Disp^{-/-}$	300 (MβCD µg/mL)	201%	
	$Disp^{-/-}$	400 (MβCD µg/mL)	358%	
	$Disp^{-/-}$	500 (MβCD µg/mL)	420%	
S3D	$Disp^{-/-}$	0 (MβCD µg/mL)	100%	0
	$Disp^{-/-}$	100 (MβCD µg/mL)	169%	92
	$Disp^{-/-}$	200 (MβCD µg/mL)	318%	93
	$Disp^{-/-}$	300 (MβCD µg/mL)	300%	39
	$Disp^{-/-}$	400 (MβCD µg/mL)	344%	129
	$Disp^{-/-}$	500 (MβCD µg/mL)	375%	91
S4A	nt Ctrl		100%	2.254
	$Disp^{-/-}$		107.8%	2.269
S4B	nt Ctrl		100%	4.482
	$Disp^{-/-}$		110.3%	2.442
S4C	nt Ctrl		100%	3.662
	$Disp^{-/-}$		110.8%	3.165
S4D	nt Ctrl		100%	3.108
	$Disp^{-/-}$		119.1%	1.800
S4E	nt Ctrl		100%	1.734
	$Disp^{-/-}$		109.7%	2.924
S4F	nt Ctrl		100%	3.349
	$Disp^{-/-}$		108.8%	1.801
	nt Ctrl	+ MβCD (0.8mg/mL)	81.51%	0.449
	$Disp^{-/-}$	+ MβCD (0.8mg/mL)	84.74%	2.085
S4G	nt Ctrl		100%	6.439
	$Disp^{-/-}$		107.2%	5.698
	nt Ctrl	+ MβCD (0.8mg/mL)	74.06%	4.110
	$Disp^{-/-}$	+ MβCD (0.8mg/mL)	76.18%	13.85
S5C	nt Ctrl	0 (HDL µg/mL)	100%	
	nt Ctrl	72 (HDL µg/mL)	235%	
	nt Ctrl	120 (HDL µg/mL)	1857%	
	$Disp^{-/-}$	0 (HDL µg/mL)	7.522%	
	$Disp^{-/-}$	72 (HDL µg/mL)	53.37%	
	$Disp^{-/-}$	120 (HDL µg/mL)	128.8%	

Journal of Cell Science • Supplementary information

Table S3. Sequences of primers used in this study.

Name	FWD Primer	REV Primer	Cloning Method
PtcΔL2 fragment 1	AAAAGCTTGGTACC ACCATGGCCTCGGC	AAGTCGACGGTGACTA TATACATGTTGTAGAA	Gibson Assembly
PtcΔL2 fragment 2	AAGTCGACGTGGGCT ACCCCTTCCTGTTCTG	AATCTAGACTAGTT GGAGCTGCTCCCCC	Gibson Assembly
DispΔL2 fragment 1	AAAAGCTTACCCGGGCAT GGCTGTGATCAGCGGAAG	AAACGCGTCTGGAA CTCAGACAGCTCCAG	Gibson Assembly
DispΔL2 fragment 2	AAACGCGTGACCTGC AGGACAGCCTTTCC	AACTCGAGTTATAGTGTTT TTATTAACAGACTCTCGC	Gibson Assembly

^{C25A}Shh	GGTGTGCCCCGGGCTGGCCG CTGGGCCCGGCAGGGGGTTT	AAACCCCCTGCCGGGCCCAGCG GCCAGCCCGGGGCACACC	Site directed mutagenesis
^{C25S}ShhN	AAAAGCTTATGCTGCTGCTG CTGGCCA	TTGGATCCCTACTCTGCTTTCAC AGAACAGTG	Cloning into pcDNA3.1
C25→S	CGGGCTGGCCAGTGGGCCC GG	CCGGGCCCACTGGCCAGCCCG	Site directed mutagenesis
Shh^{HA}	AACTCGAGATGCTGCTGCTG CTGGCC	AAACGCGTTCAGCTGGACTTGA CCGCC	Cloning into pIRES vector

References:

35. Stewart DP, Marada S, Bodeen WJ, Truong A, Sakurada SM, Pandit T, Pruett-Miller SM, Ogden SK (2018) Cleavage activates dispatched for Sonic Hedgehog ligand release. Elife 7. doi:10.7554/eLife.31678
