# Supplementary material for: Conserved cholesterol-related activities of Dispatched 1 drive Sonic hedgehog shedding from the cell membrane
Source: J Cell Sci. 2021 Aug 19;135(5):jcs258672. doi: 10.1242/jcs.258672 (PMC8403983; doi:10.1242/jcs.258672)
Supplement: Reviewer comments [file joces-135-258672_review_history.pdf]

Conserved cholesterol-related activities of Dispatched 1 drive Sonic hedgehog shedding from the cell membrane

Kristina Ehring, Dominique Manikowski, Jonas Goretzko, Jurij Froese, Fabian Gude, Petra Jakobs, Ursula Rescher, Uwe Kirchhefer and Kay Grobe
DOI: 10.1242/jcs.258672

Editor: James Olzmann

Review timeline
Original submission: 19 March 2021
Editorial decision: 27 April 2021
First revision received: 8 July 2021
Accepted: 8 July 2021

Original submission

First decision letter

MS ID#: JOCES/2021/258672

MS TITLE: Conserved cholesterol-related activities of Dispatched drive Sonic hedgehog shedding from the cell membrane

AUTHORS: Kristina Ehring, Dominique Manikowski, Jonas Goretzko, Jurij Froese, Fabian Gude, Petra Jakobs, Ursula Rescher, and Kay Grobe

ARTICLE TYPE: Research Article

We have now reached a decision on the above manuscript.

To see the reviewers' reports and a copy of this decision letter, please go to: https://submit-jcs.biologists.org and click on the 'Manuscripts with Decisions' queue in the Author Area. (Corresponding author only has access to reviews.)

As you will see, the reviewers are generally positive and highlight the high degree of interest in the observed link between dispatched / patched, cholesterol, and sonic hedgehog shedding. However, the reviewers raise some concerns that prevent me from accepting the paper at this stage. They suggest, however, that a revised version might prove acceptable, if you can address their concerns. If you think that you can deal satisfactorily with the criticisms on revision, I would be pleased to see a revised manuscript. We would then return it to the reviewers.

We are aware that you may be experiencing disruption to the normal running of your lab that makes experimental revisions challenging. If it would be helpful, we encourage you to contact us to discuss your revision in greater detail. Please send us a point-by-point response indicating where you are able to address concerns raised (either experimentally or by changes to the text) and where you will not be able to do so within the normal timeframe of a revision. We will then provide further guidance. Please also note that we are happy to extend revision timeframes as necessary.

Please ensure that you clearly highlight all changes made in the revised manuscript. Please avoid using 'Tracked changes' in Word files as these are lost in PDF conversion.

© 2021. Published by The Company of Biologists under the terms of the Creative Commons Attribution License (https://creativecommons.org/licenses/by/4.0/).

I should be grateful if you would also provide a point-by-point response detailing how you have dealt with the points raised by the reviewers in the 'Response to Reviewers' box. Please attend to all of the reviewers' comments. If you do not agree with any of their criticisms or suggestions please explain clearly why this is so.

Reviewer 1

Advance summary and potential significance to field

This is an interesting work on the detailed mechanism of Sonic Hedgehog (Shh) shedding. Interestingly the authors show that Dispatched extracts cholesterol from membranes thereby aiding the proteolytic release of Shh. This is an important contribution on the faction of Dispatched in the Shh pathway. In general the work is technically sound and well presented. Overall the conclusions are convincing despite some major and minor remarks to this:

Comments for the author

Major:
P5 lines 162-163: “We did not observe significantly increased Shh shedding from DispdL2 expressing Disp-/- cells (Fig. 3b, b’), indicating that the Disp L2 contributes to the process.” It is not correct to conclude a positive contribution from the absence of a significance, especially if the quantification shows certainly a trend toward increase of Shh shedding from DispdL2 expressing Disp-/- cells. And also the WB shows this. This problem can be solved by increasing the statistical power of the analysis by repeating it in independent experiments.
General statistics (throughout the manuscript): For instance Fig 3b’: “n=7 datasets from 5 independent experiments”. Does this mean that some datasets (i.e. experimental units; “n”) in the sample were not independent? Independency would be required for the statistical methods used in this work. Please explain and correct troughout.

Minor:
Figures 1 and 2: Fig 1 c shows C25AShhN, Fig 2c shows C25SShhN; is this correct? If yes; what is the rational for using different mutants (please make it clear to the general reader).
P3,line 54: “at the C-terminal” and “at the N-terminal”; should be “at the C-terminus” or “at the C-terminal end”; same for N-terminus; please consider.
P3, lines 81/82: “export into the ER” is a bit imprecise; better terminology would be “during translocation into the ER”. The cleavage of the signal peptide occurs at the extracytoplasmic site of the membrane (i.e. inside the ER).
P4,line 101: “relay”? Do you mean “release”?
P5, line 172: “One solution to the question..” better “answer to the question”? (I can solve problems and answer questions; please consider).

Reviewer 2

Advance summary and potential significance to field

This paper uses a well established essay of Disp1-mediated Shh shedding from Bosc23 cells to establish a link between Ptch1/Disp1- cholesterol distribution and Shh shedding.

Comments:

Disp1-/-: One allele is described as a 7 bp loss. What is the nature of the second allele, and is the Bosc23 cell line 2n?

The observation that Disp1tg, Ptch1tg and in particular Ptc1Delta-loop2tg rescue Shh shedding from Disp1-/- Bosc23 cells is interesting and suggest that the RND activity associated with both Ptc1 and Disp1 aids in shedding. The best interpretation of the Delta-loop2 form of Ptch1 is that it maintains its antiporter activity but cannot be inhibited by Shh. As the extreme N-terminus of Shh interacts

© 2021. Published by The Company of Biologists under the terms of the Creative Commons Attribution License (https://creativecommons.org/licenses/by/4.0/).

with Ptch1 in a manner that would prevent proton entry and thus antiporter activity. It would be informative to test if this lipdated Shh fragment can prevent Shh shedding. Alternatively, all RNDs rely on a typical glutamic acid in TM4 that is required for antiporter activity. Ptch1 and Disp1 mutants at that residue should not cause Shh shedding if mediated via the antiporter activity. Disp2 lacks this glu residue and should not mediate shedding either. Implicating this catalytic residue would much strengthen (or dismiss) the proposed link between Ptch/Disp cholesterol antiporter activity and Shh shedding

Although the cyclodextrin results are clear, and alternate interpretation would be that for example cyclodextrin solubilizes Shh by protecting one or both of its lipidations. Alternate ways of depleting cholesterol (e.g 7DHCR mutants, statins, lipid depleted medium or a combination thereof) should be used to support the direct cholesterol concentration/shedding argument. Along the same lines, making the RND NPC1 null should significantly change cellular cholesterol distribution, and likely alter Shh shedding.

Comments for the author

Some very simple experiments would greatly strengthen the paper in my opinion.

First revision

Author response to reviewers' comments

Reply to reviewer comments

We appreciate the positive comments and constructive suggestions for improving the manuscript. Following both reviewer suggestions, we added additional *in vitro* experiments to the revised manuscript and improved data analysis where necessary. Please find our answers to reviewer comments below:

Reviewer 1:

Major points:

Reviewer 1 suggested additional experiments to increase the statistical power of DispΔL2- mediated Shh solubilization from the surface of cells that express both proteins (Former and revised Fig. 3b,b'). We conducted five additional independent experiments but still fail to observe statistically significant increases in Shh release. From this we conclude that the second extracellular Disp (L2) loop contributes to Shh shedding. This is now clearly stated in the manuscript (line 187ff).

Following the first reviewer's suggestion, we checked and, where necessary corrected the statistical methodology throughout this paper. It was alo double-checked by an expert in statistics at this University (Prof. Andreas Heuer, Institute of Physical Chemistry, University of Münster). Moreover, we now clearly state that all experimental data (including what was formerly described as "datasets") were derived independently (line 462ff).

Minor points:

1) Both, C25S and C25A mutagenesis are known to impair N-terminal Shh palmitoylation and result in the same functional loss of Hh biofunction, both in vitro and in vivo. This is now described in the revised text (line 143) and the relevant reference is now added (Hardy and Resh, 2012).

2) All other minor suggestions of this reviewer are also addressed and included in the revised manuscript.

© 2021. Published by The Company of Biologists under the terms of the Creative Commons Attribution License (https://creativecommons.org/licenses/by/4.0/).

Reviewer 2:

Major points:

Reviewer 2 raised the important possibility that Bosc23 cells are not 2n, with important implications on our CRISPR/Cas9 mediated Disp inactivation. Indeed, Bosc23 cells are derived from HEK293 cells, and the modal chromosome number of this cell line is 64. This is now stated in the materials section (line 329ff). Despite this obstacle, we were able to isolate two independent Disp$^{-/-}$ lines carrying mutations in all three Disp loci, and in one case (that we used throughout our presented work) it was always the same mutation, as described (334ff). To be absolutely sure Disp$^{-/-}$ cells were truly null, we a) directly sequenced the PCR-amplified target region, b) sequenced ten cloned PCR products obtained from each candidate cell line to confirm Disp inactivation, c) compared two independently derived Disp$^{-/-}$ lines, which behaved identically, and c) used protein blotting to confirm the null genotype (as shown in Fig. S1).

Reviewer 2 also remarked that some very simple experiments would greatly improve the paper and suggested several alternate ways to strengthen the link between Disp-mediated cholesterol export and shedding. Thus, to test our model of membrane cholesterol export by Disp, we compared [^{3}H]-cholesterol export from Disp-deficient cells with [^{3}H]-cholesterol export from control cells (now shown in Figure 5e, results described in lines 103-105, 233- 242, 422ff). This experiment demonstrated [^{3}H]-cholesterol export from cells that express endogenous Disp into the media, and that [^{3}H]-cholesterol export from Disp-deficient cells (that are impaired in their capacity to shed Shh) was reduced in three independent assays.
This was despite the fact that Disp-deficient cells contained increased amounts of [^{3}H]- cholesterol in their membranes, in full support of our previously shown AMPLEX total cholesterol quantification assays (Fig. 5c,d). The observation is also fully in line with postulated RND-transporter function for small molecules and the presence of a sterol sensing domain in Disp. In this experiment, M¤CD extracted similar cholesterol amounts from the plasma membrane of both cell lines, supporting the proposed link between cholesterol extraction and Shh shedding (Fig. 5e). Finally, we would like to note that the alternative possibility of dual-lipidated Shh extraction by M¤CD, as suggested by this reviewer, is not likely because M¤CD-solubilized Shh was always found to be truncated (which is not to be expected if the morphogen is merely extracted, see Fig. 1). This is now also stated in the revised manuscript (line 218).

Another experiment suggested by the reviewer was to assess the capacity of Disp antiporter-deficient variants to release Shh via shedding, because in contrast to transgenic Disp^{WT} that had already been tested in our original work, we would expect that antiporter-deficient variants do not increase Shh shedding over control levels. We cloned two Disp variants that were previously established to be biologically inactive, Disp^{AAA} and Disp2 (Ma et al., 2002), and conducted the requested assays. Although the obtained results (shown below) match our expectations, we were not able to confirm comparable Disp^{WT}/mutated Disp protein expression levels on Westerns, despite the use of HA-tags. This is likely a consequence of required Shh+Hhat, Scube2 and Disp^{WT}/Disp2/Disp^{AAA} co-transfections, and the resulting low expression levels of the Disp constructs. Probably for the same reason, we could also observe reduced levels of N-terminal lipidation and processing in some assays. Ultimately, we resorted to confirm similar transcription levels of Disp^{WT} and both mutant variants by qRT-PCR. Because of the continued technical problem to quantify the relative protein amounts in our assays, we still consider our results too preliminary and would like to not include them in the revised publication. Instead, we plan to investigate their capacity to export [^{3}H]-cholesterol and to compare these capacities with their (impaired) abilities to enhance Hh shedding as better proof in the future.

© 2021. Published by The Company of Biologists under the terms of the Creative Commons Attribution License (https://creativecommons.org/licenses/by/4.0/).

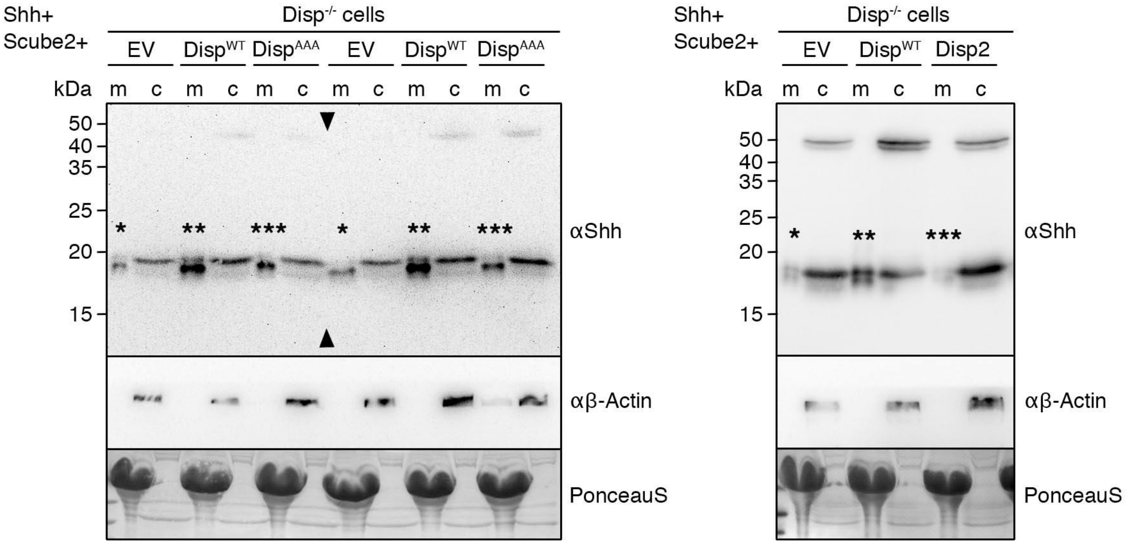

If compared to Shh release in the absence of DispWT or the Disp variants (*), Disp co-transfection increases Shh shedding and release (**, note the size shift), and the activities of DispAAA and Disp2 are reduced (***), as expected. Left: Two independent assays comparing DispWT-and DispAAA-mediated Shh release from the surface of cells co-transfected with Shh, Hhat, and Scube2. Right: Two independent assays comparing DispWT-and Disp2-mediated Shh release from the surface of cells co-transfected with Shh, Hhat, and Scube2. qRT-PCR was used to confirm similar transcription of DispWT and DispAAA; in the assay shown on the right, Disp2 transcrips were 3 times more abundant than DispWT transcripts.

Ma, Y., Erkner, A., Gong, R., Yao, S., Taipale, J., Basler, K. and Beachy, P. A. (2002). Hedgehog-mediated patterning of the mammalian embryo requires transporter-like function of dispatched. *Cell* **111**, 63-75.

Second decision letter

MS ID#: JOCES/2021/258672

MS TITLE: Conserved cholesterol-related activities of Dispatched drive Sonic hedgehog shedding from the cell membrane

AUTHORS: Kristina Ehring, Dominique Manikowski, Jonas Goretzko, Jurij Froese, Fabian Gude, Petra Jakobs, Ursula Rescher, Uwe Kirchhefer, and Kay Grobe

ARTICLE TYPE: Research Article

I am happy to tell you that your manuscript has been accepted for publication in Journal of Cell Science, pending standard ethics checks. Thank you for submitting this interesting manuscript to JCS!

© 2021. Published by The Company of Biologists under the terms of the Creative Commons Attribution License (https://creativecommons.org/licenses/by/4.0/).
